# Single-Cell Transcriptomics Shows Cellular Heterogeneity, Intercellular Communication, and Extracellular Matrix Remodeling in Corneal Fibrosis In Vivo

**DOI:** 10.1167/iovs.66.13.48

**Published:** 2025-10-28

**Authors:** Rajnish Kumar, Nishant R. Sinha, Maxwell T. Jeffrey, Alexandria C. Hofmann, Rajiv R. Mohan

**Affiliations:** 1 Harry S. Truman Memorial Veterans’ Hospital, Columbia, Missouri, United States; 2 Departments of Veterinary Medicine & Surgery and Pathobiology and Integrative Biomedical Sciences, College of Veterinary Medicine, University of Missouri, Columbia, Missouri, United States; 3 Mason Eye Institute, School of Medicine, University of Missouri, Columbia, Missouri, United States

**Keywords:** cellular heterogeneity, fibrosis, single-cell RNA sequencing, stromal remodeling, wound healing

## Abstract

**Purpose:**

Corneal fibrosis is a common clinical condition post ocular trauma/infection affecting 7% of world's population. This study characterized fibroblast heterogeneity, cellular trajectories, intercellular communications, and extracellular matrix (ECM) protein remodeling in fibrotic cornea in vivo via single-cell RNA sequencing (scRNA-seq).

**Methods:**

Naïve and alkali-injured fibrotic corneas of New Zealand White rabbits were obtained under approved institutional animal use and care protocol. Multimodal in vivo eye imaging, single-cell RNA-sequencing, hematoxylin and eosin staining, and immunofluorescence analyzed corneas. Unsupervised clustering, subclustering of ECM remodeling cells, trajectory inference, CellChat-based intercellular communication mapping, and R-programming–generated transcriptomic atlas.

**Results:**

Fourteen transcriptionally distinct cell clusters were identified via canonical marker genes. Basal epithelium and ECM remodeling clusters acted as communication hubs whereas differentiated epithelium had limited participation. Subclustering the ECM remodeling niche identified four distinct stromal cell populations, quiescent-keratocytes, activated-keratocytes, progenitor-like-keratocytes, and myofibroblasts. Transcriptional trajectories organized these cellular states into a bifurcating continuum; one aligned to fibroblast activation and myofibroblast formation whereas the other aligned to quiescent-keratocytes or progenitor-like-keratocytes. Eight pathways (MIF, NECTIN3, HGFα, POSTN, NAMPT, TWEAK, EPH, and VEGFC) demonstrated strong ligand-receptor connectivity and their protein expression corroborated predicted signaling. Stage-specific enrichment revealed temporal dynamics within ECM-remodeling cells and continuous landscape of stromal subpopulations from quiescent-keratocytes to proliferative-fibroblasts/myofibroblasts and progenitor-like-cells followed by late dominance of ECM organization, adhesion, and collagen biosynthesis. The progression trajectory appeared bidirectional along pseudotime.

**Conclusions:**

This study provides an integrative transcriptomic framework of stromal cell heterogeneity, intercellular signaling, and ECM remodeling trajectories in rabbit cornea in vivo and advances mechanistic understanding of corneal repair.

Corneal fibrosis is a pathological consequence of aberrant tissue repair after ocular trauma or infection. It commonly arises because of persistent inflammation and deviations in a tightly regulated wound healing process. After insult, resident corneal stromal keratocytes are activated to fibroblasts and become myofibroblasts acquiring a contractile phenotype and synthesizing/secreting cytokines and large amounts of extracellular matrix (ECM) components to facilitate tissue repair and remodeling. These processes are essential to restore corneal transparency and vision.^1^

Fibrosis is a highly regulated and dynamic process involving coordinated interactions between the stromal, immune, and vascular compartments in the cornea. Recent lineage tracing and single-cell transcriptomic studies have revealed considerable cellular heterogeneity within fibrotic tissues, identifying distinct fibroblast subsets and other cell types involved in ECM remodeling and wound healing.^2^^–^^8^ However, a comprehensive understanding of the lineage relationships, temporal progression, and intercellular signaling that govern these responses remains limited. Elucidating these dynamic cellular trajectories and communication networks holds potential for identifying critical regulators and therapeutic targets that control fibrosis progression and ECM remodeling across tissues. Cornea being an avascular and immune-privileged tissue, offers a unique model to study the cellular transition and fibrosis development in a tissue.^9^ The avascularity of the central cornea minimizes direct vascular confounders while still permitting immune infiltration after injury, providing a uniquely tractable milieu to study fibroblast transitions and matrix remodeling. Cornea has cellular heterogeneity because of its quarantined microenvironment, accessibility, and distinctive collagen architecture and three major cell layers, epithelium, stroma, and endothelium.^10^ Owing to the limited interactions with vascular components, the cornea presents a clean and controlled microenvironment for studying fibroblast dynamics, stromal cell activation, myofibroblast biology, and ECM remodeling processes. Furthermore, corneas allow visual monitoring of fibrotic state, as well as qualitative and quantitative measurements of changes in the cellular phenotypes and density/population via multimodal two- and three-dimensional eye imaging in live animals in vivo.^11^

Chemical injury, particularly alkali burns, is the physiologically relevant and reproducible in vivo model for studying fibrosis and wound healing events in the cornea. Alkali injury accounts for the majority of ocular trauma globally and causes a range of morbidities because of its ability to rapidly penetrate into the cornea causing widespread cellular disruption, ECM damage, and glycosaminoglycans degradation. Corneal alkali burns, among the most severe ocular traumas, trigger epithelial breakdown, immune activation, and stromal fibroblast reprogramming—hallmarks of fibrotic remodeling observed across diverse tissues.^12^^–^^16^ The subsequent cascade of fibrosis and pathological angiogenesis presents a significant clinical challenge, often culminating in vision impairment. The fibrotic cornea of rabbits closely recapitulates histopathological and molecular features observed in human cornea, which makes it a relevant model for studying fibrotic mechanisms and identifying effective therapeutic targets.^17^^–^^20^

Comprehensive profiling of cell states, lineage trajectories, and intercellular signaling within complex tissues via single cell RNA-sequencing (scRNA-seq) has enabled high-resolution dissection of dynamic cellular responses during fibrosis and tissue remodeling.^21^^–^^25^ By capturing transcriptomic changes at single-cell resolution, scRNA-seq provides a powerful platform for investigating fibroblast heterogeneity, cellular plasticity, and molecular signaling networks involved in wound healing. In this study, we performed scRNA-seq analysis using R programming to generate a detailed cellular atlas of fibrotic rabbit corneas at 14 days after alkali injury, a timepoint coinciding with peak stromal remodeling, to highlight the transcriptional landscape of fibrosis. This sought to identify critical regulators of fibroblast activation, stromal cell transitions, ECM remodeling, myofibroblast contributions, and role of ligand-receptor-mediated cellular communication in vivo using naïve and alkali-injured rabbit corneas. Furthermore, through quantitative analysis and statistical computing with R packages, involving unsupervised clustering, pseudotime trajectory inference, and regulatory network analysis, we aimed to identify stromal cell-state diversity and uncover progressive transitions that drive fibrotic scarring in the cornea. The insights gained from this study regarding the regulation of fibroblasts, myofibroblasts, and ECM remodeling dynamics may contribute to a broader understanding of fibrosis across tissue types.

## Methods

### Anesthesia and Alkali-Induced Corneal Wounding in Rabbits

The animal procedures in this study were conducted with approval from the Institutional Animal Care and Use Committee at the University of Missouri and the Harry S. Truman Memorial Veterans’ Hospital. All experiments adhered to the guidelines outlined in the ARVO Statement for the Use of Animals in Ophthalmic and Vision Research. The study used six New Zealand white rabbits (Charles River, Wilmington, MA, USA), each weighing between 2 and 3 kg. To induce corneal fibrosis via alkali wounding, the rabbits were anesthetized via intramuscular injection of ketamine hydrochloride (50 mg/kg; JHP Pharmaceuticals, LLC, Rochester, MI, USA) and xylazine hydrochloride (10 mg/kg; XylaMed, Bimeda Inc., IL, USA). Periodic clinical eye examinations were performed via slit-lamp microscope (SL-15; Kowa Optimed, Torrance, CA, USA), ophthalmic stereo microscope (Leica DM 4000B; Leica, Wetzlar, Germany) equipped with a digital camera (SpotCamRT KE; Diagnostic Instruments, Sterling Heights, MI, USA), and Pentacam HR (Oculus GmbH, Arlington, WA, USA). Applanation tonometry (Tono-Pen AVIA; Reichert Technologies, Depew, NY, USA) measured intraocular pressure. Before any procedure, local anesthesia was provided by two drops of topical ophthalmic proparacaine hydrochloride (0.5%; Alcon, Fort Worth, TX, USA). Corneal fibrosis was produced in the right eye of three rabbits whereas the other three rabbits remained unwounded and served as naïve control. The rabbits were anesthetized, and topical anesthesia was applied before a 1 M sodium hydroxide solution was applied to the central cornea for 30 seconds via 8 mm filter paper under a surgical microscope (Leica). The eye was rinsed with plenty of sterile ophthalmic balanced salt solution to eliminate any remaining alkali and monitored for 14 days.

### Multimodal Clinical Corneal Imaging

Ophthalmic Slit-lamp, Stereo-, and Pentacam biomicroscopy acquired corneal status before injury and on days 1, 3, 7, and 14 after injury. Scheimpflug tomography captured 25 images/eye per session; scans with quality scores < 95% were repeated (Pentacam HR, Oculus GmbH software v1.28r). Densitometry (0–100 gray units), pachymetry and simulated keratometry maps were exported as CSV files for downstream statistics. Intraocular pressure was measured in triplicate between 09:00–11:00 h; readings with standard deviations > 1 mm Hg were discarded.

### Single-Cell Lysate Preparation

Rabbit corneas were collected and processed for single-cell lysate preparation following our laboratory-optimized enzymatic digestion protocol to ensure effective dissociation while preserving cellular integrity. In brief, injured and naïve corneas were harvested using surgical blade and placed in Falcon Round-Bottom Polystyrene Tubes (Thermo Fisher Scientific, Waltham, MA, USA). A circumferential incision was made parallel to the limbus, leaving a ∼1 mm peripheral rim of limbus attached to the sclera, and the cornea (epithelium, stroma, and endothelium) was excised without adjacent sclera/limbus. The central corneal button (epithelium, stroma, endothelium) was collected for cells isolation. Each rabbit cornea was sectioned into 1/8th pieces, and a total of four pieces (equivalent to half a cornea) per tube were used for enzymatic digestion. The tissues were submerged in 300 µL of digestion buffer and maintained at 4°C during the initial processing. To ensure complete dissociation into a single-cell suspension, a digestion buffer was prepared containing 2.5 mg of Liberase TL (Cat no. 17101015; Thermo Fisher Scientific), 2 mg of deoxyribonuclease I, bovine pancreas (Cat no. AAJ62229MB; Thermo Fisher Scientific), and 1 mL of incomplete RPMI 1640 medium w/L-glutamine (Cat no. 11875119; Thermo Fisher Scientific). The corneal tissue within the tubes was finely chopped via sterile scissors while submerged in digestion buffer to increase enzymatic exposure. The tubes were then incubated in a heat block with a shaker set to 400 rpm at 37° C for 30 minutes. After incubation, a 200 µL pipette tip was used to disrupt any remaining tissue clumps, with additional agitation every five minutes was conducted until full dissociation was achieved.

To halt enzymatic digestion and stabilize the cells, an equal volume of 100% fetal bovine serum (FBS), heat-inactivated (Cat no. 10082147; Thermo Fisher Scientific), was added to the Falcon tubes (300 µL of FBS per 300 µL of digestion buffer) and mixed thoroughly. The solution was pipetted repeatedly to further dissociate the cells before being filtered through a 70 µm Falcon Cell Strainer (Cat no. 08-771-2; Thermo Fisher Scientific) into 15 mL Falcon tubes.

The filtered cell suspension was subjected to three sequential washes with 3 mL of fluorescence-activated cell sorting buffer (phosphate-buffered saline solution + 2% FBS) to remove enzymatic residues and debris. The samples were subsequently spun in a centrifuge at 200*g* (1000 rpm) for five minutes, with the brake/deceleration set to 1 to prevent cell loss. The supernatant was carefully removed via a pipette (vacuum removal was avoided to prevent cell disruption). Finally, the cell pellet was resuspended in 300 µL of fluorescence-activated cell sorting buffer, and volume was adjusted according to the cell concentration requirements. This optimized protocol facilitated the preparation of high-quality single-cell lysates suitable for downstream scRNA-seq.

### Library Preparation and Sequencing

The scRNA-seq was conducted via the 10x Genomics Chromium platform following standard protocols for single-cell library preparation. The 10× Genomics single-cell 3′ library preparation was performed by the University of Missouri, Columbia Genomics Technology Core at the Bond Life Sciences Center. Sequencing was conducted on an Illumina NovaSeq X Plus instrument, generating 100 base-pair, paired-end reads. The expected sequencing depth was ∼50,000 read pairs per cell per library, optimized for 3′ RNA sequencing, with a target of 10,000 single-cell transcriptomes per sample to ensure high coverage and resolution. The scRNA-seq libraries were constructed via 10x Genomics Chromium Chip technology, with precise library preparation to ensure optimal cell recovery, minimal background noise, and accurate unique molecular identifier (UMI) processing. All sequencing and data preprocessing were performed at the University of Missouri Genomics Core Facility, with rigorous data quality control at each step.

### Raw Data Preprocessing and Alignment

The raw sequencing data were processed via Cell Ranger v7.0.0, a suite developed by 10x Genomics for single-cell data analysis. This involved demultiplexing the sequencing reads, barcode processing, UMI counting, and alignment to the rabbit reference genome (GCF_009806435.1_UM_NZW_1.0). The pipeline generated a gene‒cell‒barcode matrix for each sample, retaining only high-confidence reads that were valid barcodes, UMIs, and confidently mapped to exonic regions.

### Quality Control and Filtering Criteria

To ensure data quality, stringent filtering criteria were applied. Cells with fewer than 1000 or more than 10,000 detected genes were removed, and cells with >10% mitochondrial transcript content were excluded. The cell viability before chip loading was 92% ± 3%, as determined by acridine-orange/propidium-iodide staining on a Countess II automated cell counter. Putative doublets were identified and removed with DoubletFinder v2.0 (expected rate = 6%, pN = 0.25, pK = 0.01; chosen via parameter sweep). Only confidently mapped, non-PCR-duplicate reads with valid barcodes and UMIs were retained to construct the final gene–cell matrix.

### Data Normalization and Processing

The Seurat R package v4.0.4 was used for downstream analysis. The data were log-normalized via the "LogNormalize" function with a scaling factor of 10,000. Highly variable genes were identified via "FindVariableFeatures", and the data were scaled, whereas mitochondrial gene expression was regressed out via "ScaleData." This step ensured that mitochondrial expression did not dominate downstream clustering and classification.

### Data Integration, Batch Correction, and Group Merging

Seurat v4.0.4’s reciprocal principal component analysis (PCA) integration workflow was run on the six individual, QC-filtered objects using 2000 highly variable genes (dims = 1–20; k.anchor = 5) to identify cross-sample anchors. After batch correction, the three alkali-burn (AB) corneas and three naïve corneas were collapsed into two biological groups, yielding a single, integrated Seurat object that served as the input for downstream PCA)/ uniform manifold approximation and projection (UMAP) embedding, clustering, and AB-versus-Naïve differential expression analyses.

### Dimensionality Reduction and Clustering

Dimensionality reduction was performed with PCA (top 20 PCs). “FindNeighbors” and “FindClusters” were run with a resolution range of 0.1–0.9, and clusters were visualized by UMAP. The ECM-remodeling parent cluster was subsequently reclustered at resolution = 0.4 (dims = 1–15) to generate four transcriptionally distinct stromal subclusters. A comprehensive map of the barcodes to the original sample identities and cluster assignments is provided in Supplementary Table S1.

### Cell-Type Annotation

Cluster identities were assigned by (i) manual inspection of canonical markers (Supplementary Table S2) and (ii) automated comparison with the CellTypist vertebrate ocular atlas (model v1.3; probability cutoff = 0.25). Manual curation overrode low-probability calls; ambiguous calls were left unassigned. Conflicts were resolved by cross-checking with marker enrichment scores.

### Marker Gene Identification and Differential Expression Analysis

Cluster-specific marker genes were identified via the "FindAllMarkers" function, which applies a stringent threshold for differential expression analysis. DEGs were identified for each cluster and annotated on the basis of known marker genes. These DEGs were further analyzed to assess their biological relevance, cluster identity, and functional implications.

### Cell-Cell Communication Inference

CellChat v1.6.1 with the “rabbit ortholog” ligand–receptor database (downloaded February 15, 2025) was used to analyze intercellular signaling. Interactions with probability <0.05 after 1000 permutations were retained, and log10-scaled probabilities were used to determine edge widths.

### Trajectory and Pseudotime Analysis

Lineages were reconstructed with Slingshot v2.10.0 via UMAP embedding, with quiescent keratocytes (cluster 0) designated as the root. TradeSeq v1.15.1 (knots = 6, nGenes = 4,000) was used to model gene expression dynamics; genes with a Wald test false discovery rate < 0.05 were considered pseudotime associated and then grouped into modules with k-means (k = 5). Diffusion components were additionally calculated with the destiny package v3.9.0 (k = 50 nearest neighbors); the first two diffusion axes were plotted to visualize the fibroblast continuum.

### Functional Enrichment Analysis

Differentially expressed gene lists were converted to human orthologs via biomaRt (Ensembl 111). Because rabbit–human mappings are not one-to-one for all genes, enrichment was interpreted with this limitation. Where available, rabbit-native annotations were cross-checked to confirm concordant pathway signals. clusterProfiler v4.10.1 was used for Gene Ontology (GO) (BP), Reactome, and Kyoto Encyclopedia of Genes and Genomes (KEGG) enrichment (universe = all expressed genes; *P*_adj_ < 0.05, Benjamini–Hochberg). Dot plots were generated with ggplot2 v3.5.2.

### Histology and Immunofluorescence

Corneas were fixed in 4% paraformaldehyde at 4°C for four hours, paraffin-embedded, and sectioned at a thickness of 5 µm. Hematoxylin and eosin (H&E) staining was performed according to standard protocols. For immunofluorescence, the tissue sections were deparaffinized, subjected to citrate-based antigen retrieval (pH 6.0, 95°C, 20 minutes), blocked in 5% BSA, and incubated overnight at 4°C with primary antibodies targeting the following proteins: α-smooth muscle actin (α-SMA) (1:300; Abcam Inc., Waltham, MA, USA; Cat no. AB322917), PDGFA (1:100; Santa Cruz Biotechnology, Santa Cruz, CA, USA; Cat no. sc-9974), vascular endothelial growth factor-A (VEGFA) (1:100; Santa Cruz Biotechnology; Cat no. sc-365578), transforming growth factor-β1 (TGFβ1) (1:100; Santa Cruz Biotechnology; Cat no. sc-130348), TGFβ2 (1:100; Santa Cruz Biotechnology; Cat no. sc-374659), ephrin type-A receptor-5 (1:100; Santa Cruz Biotechnology; Cat no. sc-517242), VEGFC (1:100; Santa Cruz Biotechnology; Cat no. sc-374628), nectin cell adhesion molecule-3 (1:100; Santa Cruz Biotechnology; Cat no. sc-271611), collagen type-I α1 chain (COL1A1) (1:100; Santa Cruz Biotechnology; Cat no. sc-293182), junctional adhesion molecule-A (JAMA) (1:100; Santa Cruz Biotechnology; Cat no. sc-53623), macrophage migration inhibitory factor (MIF) (1:100; Santa Cruz Biotechnology; Cat no. sc-271631), LAMININ5 (1:100; Santa Cruz Biotechnology; Cat no. sc-13587), hepatocyte growth factor-α chain (HGFα) (1:100; Santa Cruz Biotechnology; Cat no. sc-374422), TNF-like weak inducer of apoptosis (TWEAK) (1:100; Santa Cruz Biotechnology; Cat no. sc-56248), nicotinamide phosphoribosyltransferase (NAMPT) (1:100; Santa Cruz Biotechnology; Cat no. sc-393444), and periostin (POSTN) (1:100; Santa Cruz Biotechnology; Cat no. sc-398631). After primary incubation, appropriate secondary antibodies were applied for one hour at room temperature: Alexa Fluor 594-conjugated goat anti-mouse IgG (1:500; Invitrogen Life Technologies, Carlsbad, CA, USA; Cat no. A48288), Alexa Fluor 488-conjugated goat anti-mouse IgG (1:500; Invitrogen Life Technologies; Cat no. A48286), or Alexa Fluor 594-conjugated goat anti-chicken IgG (1:500; Invitrogen Life Technologies; Cat no. A11042), depending on the primary antibody host. Nuclei were counterstained with DAPI. Images were captured via a Leica DMi8 fluorescence microscope (300 ms exposure time).

### Statistical Analysis and Data Interpretation

All the statistical analyses were conducted via R (v4.2.2) and GraphPad Prism (v9.5.1). The scRNA-seq differential gene expression analyses were performed via Wilcoxon rank-sum tests as implemented in Seurat's FindMarkers function, with Benjamini–Hochberg correction for multiple testing (adjusted *P* value < 0.05 was considered significant). Pseudotime-associated genes were selected via generalized additive models and Wald tests in tradeSeq, with false discovery rate < 0.05. Pathway enrichment was performed via hypergeometric tests with multiple testing correction (*P*_adj_ < 0.05). All bar graph values represent the mean ± SEM unless otherwise specified. No statistical methods were used to predetermine sample size; however, biological replicates (*n* = 3 per group) and stringent quality controls were used throughout.

## Results

### Corneal Fibrosis and Structural Alterations After Injury

**Figure 1.**
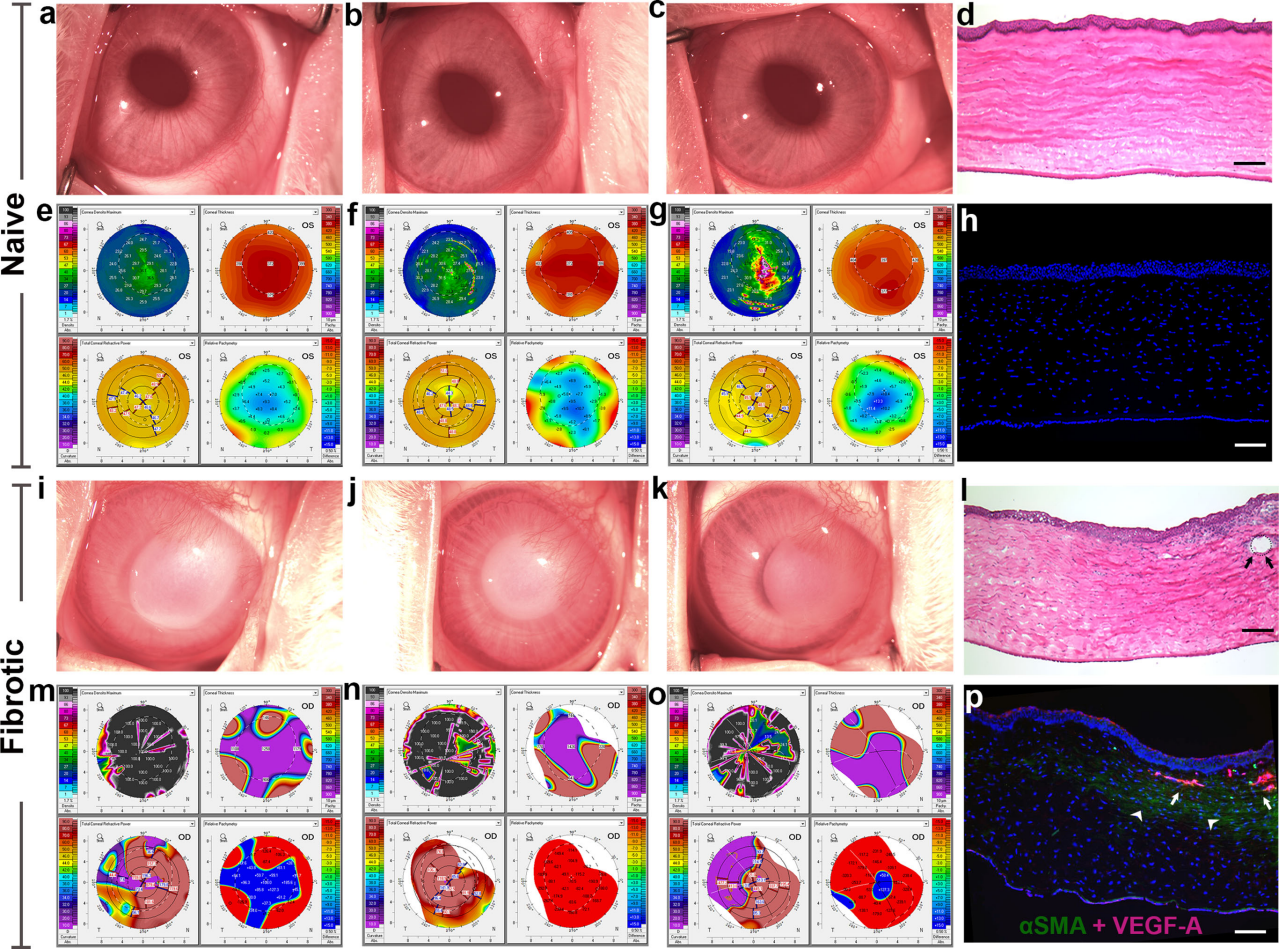
Imaging, histology, and protein expression in corneal fibrosis on day 14 after alkali injury. **(a–c)** Stereomicroscopy images of naïve eyes with clear and smooth corneas. **(i–k)** Eyes with fibrotic corneal opacity and surface irregularity. **(e–g)** Pentacam tomography of naïve eyes showing uniform corneal density, central thickness, curvature, and pachymetry. **(m–o)** Eyes with fibrotic corneas showing increased corneal density, thickening, curvature irregularity, and disrupted pachymetry. **(d, l)** H&E images showing an organized epithelium, stromal lamellae, and endothelium in naïve tissue and lamellar disarray with edema, inflammatory infiltration, and neovascular invasion in fibrotic tissue (*arrow*). **(h, p)** Immunofluorescence for α-SMA (*green*) and VEGFA (*red*) showing minimal stromal expression in naïve tissue and stromal α-SMA-positive myofibroblasts (*arrowheads*) with increased stromal VEGFA expression (*arrows*) in fibrotic tissue. Nuclei were counterstained with DAPI; *n* = 3 biological replicates per group. *Scale* *bar*: 100 µm.

An ophthalmic stereomicroscope revealed optically clear and moist smooth surfaces in all naïve corneas (Figs. 1a–c), whereas fibrotic corneas presented diffuse stromal haze, surface irregularities, and opacification, the hallmarks of fibrosis (Figs. 1i–k). Pentacam-assisted corneal tomography demonstrated substantial alterations in optical and biomechanical metrics postinjury. Fibrotic corneas presented increased stromal density, stromal thickening, irregular curvature, and disrupted pachymetry gradients (Figs. 1m–o), whereas naïve tissue presented uniform stromal density, central thickness, curvature, and pressure distribution (Figs. 1e–g). The fibrotic corneas exhibited stromal disorganization, edema, and neo-vessels (Fig. 1l) contrary to the naïve tissues (Fig. 1d) in H&E staining. Double immunofluorescence staining for αSMA and VEGFA confirmed fibrosis and angiogenesis, respectively. Fibrotic corneas exhibited significantly high αSMA, indicative of myofibroblast presence, and VEGFA expression (Fig. 1p), whereas no noticeable αSMA or VEGFA expression was detected naïve cornea (Fig. 1h).

### Cellular Heterogeneity

**Figure 2.**
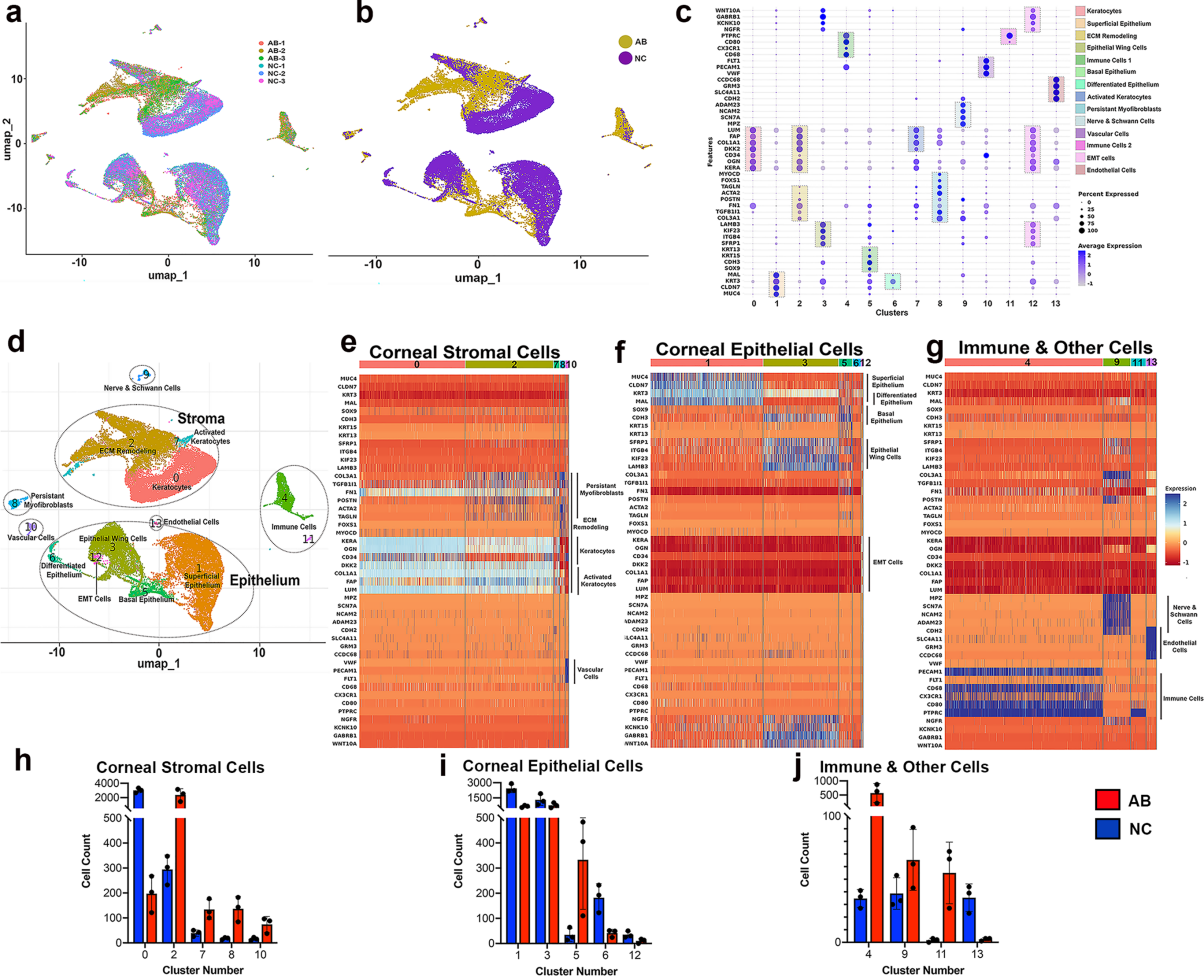
Cellular composition and fibrosis-associated transcriptional shift at single-cell resolution. **(a)** UMAP of single cells from fibrotic/alkali-burn (AB) and naïve (NC) corneas, colored according to biological replicates (AB-1, AB-2, AB-3 and NC-1, NC-2, NC-3), showing reproducibility. **(b)** UMAP colored by condition (AB *yellow* and NC *purple*) showing fibrosis-associated shifts in the cellular distribution on day 14 after injury. **(c)** Dot plot of canonical marker genes used for cell type annotation; the dot size indicates the percentage of cells expressing the gene, and the color intensity indicates average expression. **(d)** UMAP annotated with 14 transcriptionally distinct clusters (clusters 0–13) corresponding to corneal cell types. **(e–g)** Heatmaps showing scaled expression of the top marker genes within the stromal **(e)**, epithelial **(f)**, and immune/other **(g)** compartments. **(h–j)** Cell counts per cluster for the fibrotic/AB (*red*) and NC (*blue*) groups. The *bars* represent the means ± SEMs of three biological replicates per group.

A reproducible cellular landscape across biological replicates was observed in naïve and fibrotic corneas (Fig. 2a). The UMAP embeddings revealed a clear spatial separation between the cellular composition and transcriptional profiles in the fibrotic (AB) and naïve cornea samples (Fig. 2b).

Unsupervised clustering identified 14 distinct transcriptional clusters (Fig. 2d). The annotated biologically meaningful cell types via canonical marker genes included multiple stromal populations (keratocytes, activated keratocytes, ECM-remodeling cells, persistent myofibroblasts, and vascular-associated cells), epithelial subtypes (superficial, wing, basal, EMT-like, and differentiated epithelial cells), and clusters representing immune cells, endothelial cells, and nerve/Schwann cells (Fig. 2c). Scaled heatmaps of the known marker genes across the stromal (Fig. 2e), epithelial (Fig. 2f), and immune/other cellular types (Fig. 2g) corroborated the cluster identities and highlighted cell-specific transcriptional reprogramming. Supplementary Table S3 provides a full list of cluster-specific genes along with the average log2-fold change, proportion of expressing cells, adjusted p values, and predicted cellular identities. The quantitative cell counts (Figs. 2h–j) in fibrotic corneas compared to naïve corneas demonstrated substantial expansion of ECM remodeling, activated keratocytes, basal epithelial cells and immune populations in fibrotic corneas, which indicates the activation of stromal remodeling, epithelial transition, and the immune response after injury.

### Intercellular Signaling Among Corneal Cell Populations

**Figure 3.**
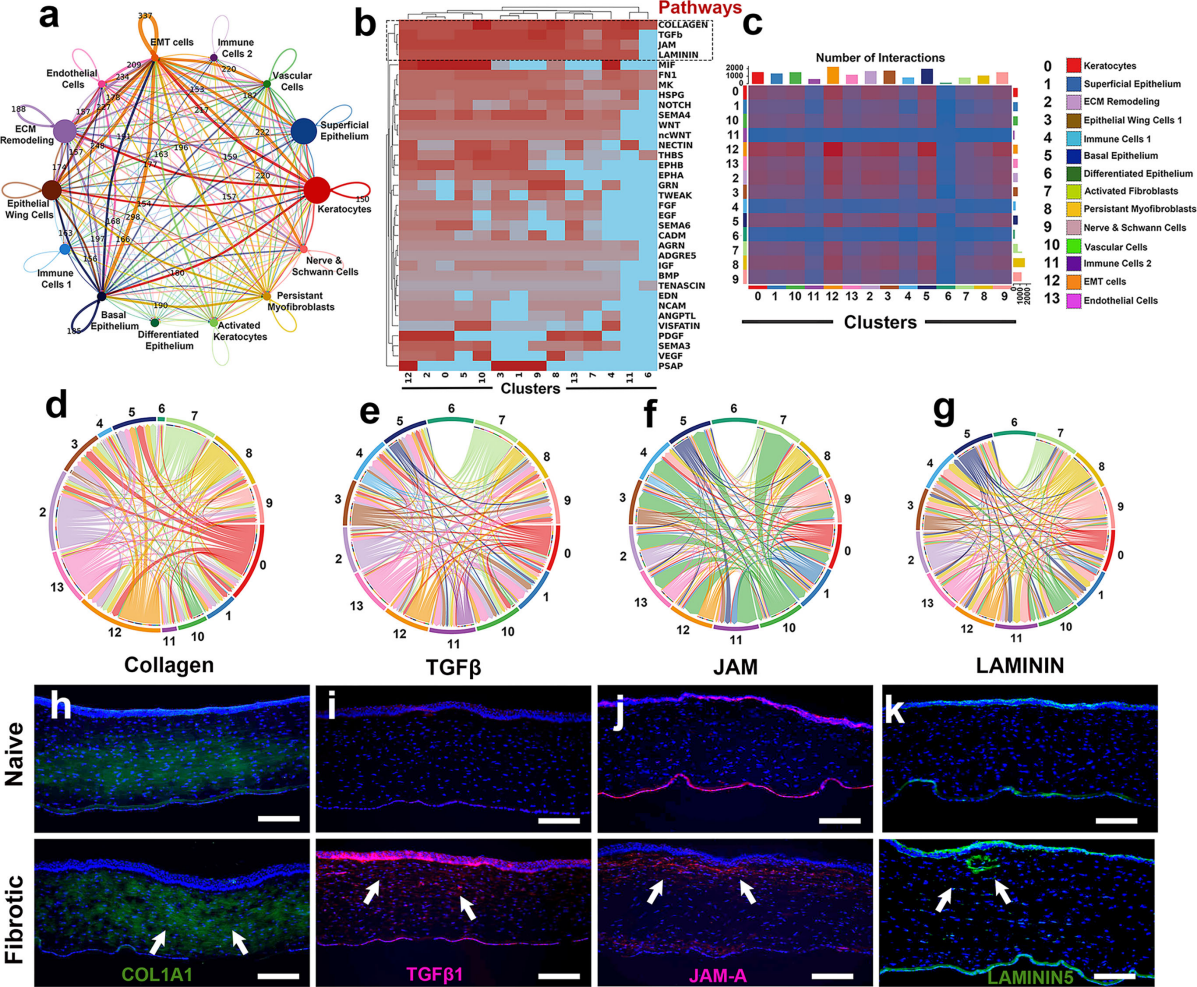
Intercellular signaling across corneal cell populations with protein validation of the top pathways associated with fibrosis. **(a)** Intercellular communication among 14 annotated cell types; node size indicates the number of cells, and edge width denotes total signaling strength. **(b)** Pathway activity by cell type highlighting the four most enriched pathways: collagen, TGFβ, JAMA, and LAMININ. **(c)** Predicted interaction counts between cluster pairs. **(d–g)** Chord for the top four pathways: collagen **(d)**, TGFβ **(e)**, JAMA **(f)**, and LAMININ **(g)**. **(h–k)** Protein-level assessment of these pathways in naïve (*top*) and fibrotic (*bottom*) tissue: increased stromal COL1A1 (*green*) **(h)** and TGFβ1 (*red*) **(i)**, and JAMA (*red*) **(j)**, and LAMININ5 (*green*) **(k)**. Nuclei were counterstained with DAPI (*blue*); *n* = 3 biological replicates per group. *Scale* *bar*: 100 µm.

The CellChat framework analysis identified global signaling network and interdirectional interactions among the 14 annotated cell types (Fig. 3a). Pathway-level inference revealed strong enrichment of ECM- and fibrosis-linked signaling pathways, including collagen, TGFβ, JAM, and LAMININ (Fig. 3b) in promoting myofibroblast differentiation and ECM deposition in cornea. Furthermore, the pairwise interaction matrix showed intense cellular signaling among these clusters (Fig. 3c). Circular chord diagrams in Figures 3d–g indicated pathway-specific communication among identified cellular clusters and the epithelial-stromal crosstalk in corneal fibrotic progression.

The expression of genes from top four dominant pathway (Fig. 3b) in naïve and fibrotic corneas were analyzed via immunofluorescence (Figs. 3h–k). The fibrotic corneal tissues showed markedly upregulated COL1A1 levels than the naïve (Fig. 3h). Likewise, expression of TGFβ1 in the fibrotic cornea was significantly higher than the naïve cornea (Fig. 3i). The expression of JAMA protein in fibrotic cornea was detected in subepithelial stromal region whereas its expression in naïve cornea was noted in epithelium and endothelium (Fig. 3j). The LAMININ5 protein, expression in naïve cornea was observed in epithelium and endothelium while in fibrotic cornea its expression was also apparent in subepithelial stromal region along with significantly upregulated levels in epithelial and endothelial layers (Fig. 3k).

### Intercellular Signaling Dynamics Among Stromal Clusters Revealed Profibrotic and Proangiogenic Remodeling

**Figure 4.**
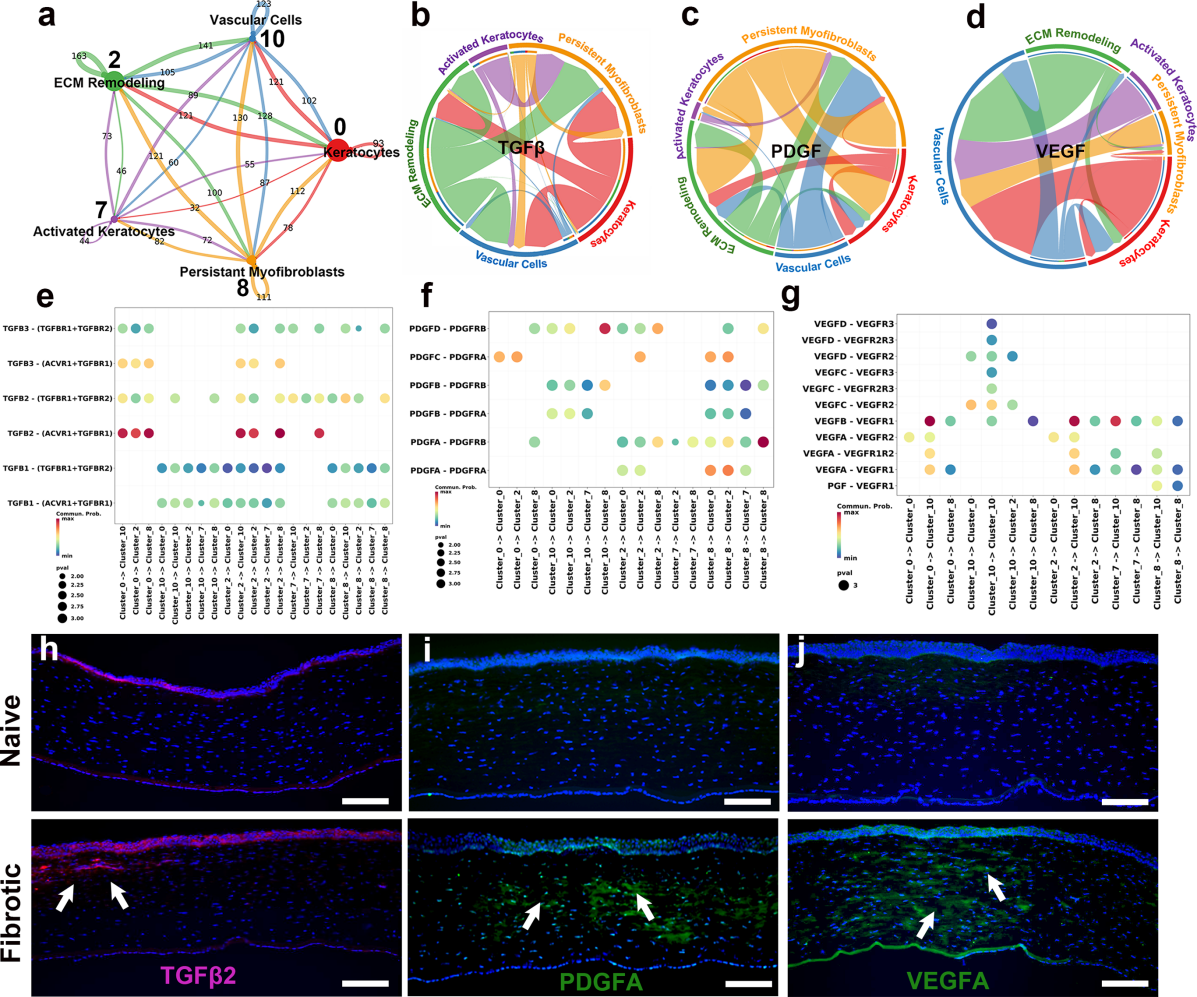
Intercellular communication among stromal clusters with key fibrotic and angiogenic axes. **(a)** Aggregated network of inferred intercellular communication among stromal clusters (keratocytes, activated keratocytes, ECM-remodeling cells, persistent myofibroblasts, and vascular cells); node size indicates the number of cells, and edge width denotes total signaling strength. **(b–d)** Chord diagrams for the TGFβ **(b)**, PDGF **(c)**, and VEGF **(d)** signaling pathways illustrating directional communication between stromal cell types. **(e–g)** Dot plots depicting individual ligand–receptor interaction probabilities for the TGFβ **(e)**, PDGF **(f)**, and VEGF **(g)** pathways. The dot size reflects the interaction strength; the color scale denotes the communication probability. Canonical pairs include TGFβ1/2–TGFBR1/2, PDGFA/B/C/D–PDGFRA/B, and VEGFA/B/C–VEGFR1/2/3. **(h–j)** Immunofluorescence validation of pathway-associated proteins in naïve (*top*) and fibrotic (*bottom*) corneas. Increased stromal expression of TGFβ2 (*red*) **(h)**, PDGFA (*green*) **(i)**, and VEGFA (*green*) **(j)** in fibrotic corneas (*white arrows*). Nuclei were counterstained with DAPI; *n* = 3 biological replicates per group. *Scale* *bar*: 100 µm.

The stromal cell clusters analysis with CellChat mapped the intercellular signaling and stromal remodeling during fibrosis. The interaction map revealed an interconnected and bidirectional network (Fig. 4a), indicating their role in corneal fibrosis and tissue repair.

Chord plots exhibited intercluster signaling pathways of TGFβ, PDGF, and VEGF (Figs. 4b–d) in corneal fibrosis. Dot plot analyses demonstrated enrichment of canonical TGFβ1/TGFβ2-TGFBR1/2 ligand-receptor pairs (Fig. 4e), PDGFA/PDGFB-PDGFRA/B axes (Fig. 4f), and VEGFA/B/C signals to VEGFR1/2/3 (Fig. 4g). This analysis showed interactions of these genes in modulation of keratocyte, myofibroblast and vascular cells and ECM-remodeling.

Whether high transcriptional activities of TGFβ2, PDGFA, and VEGFA translate to protein level was evaluated with immunofluorescence (Figs. 4h-j). TGFβ2 expression was markedly greater in the epithelium and anterior stroma of fibrotic corneas than the naïve corneas (Fig. 4h). The PDGFA (Fig. 4i) and VEGFA (Fig. 4j) expression was notably higher in the entire fibrotic cornea compared to the naïve cornea. These analyses displayed dominant role of TGFβ2, PDGFA, and VEGFA in corneal tissue remodeling.

### Subclustering Revealed Functional Heterogeneity Within the ECM-Remodeling Cluster

**Figure 5.**
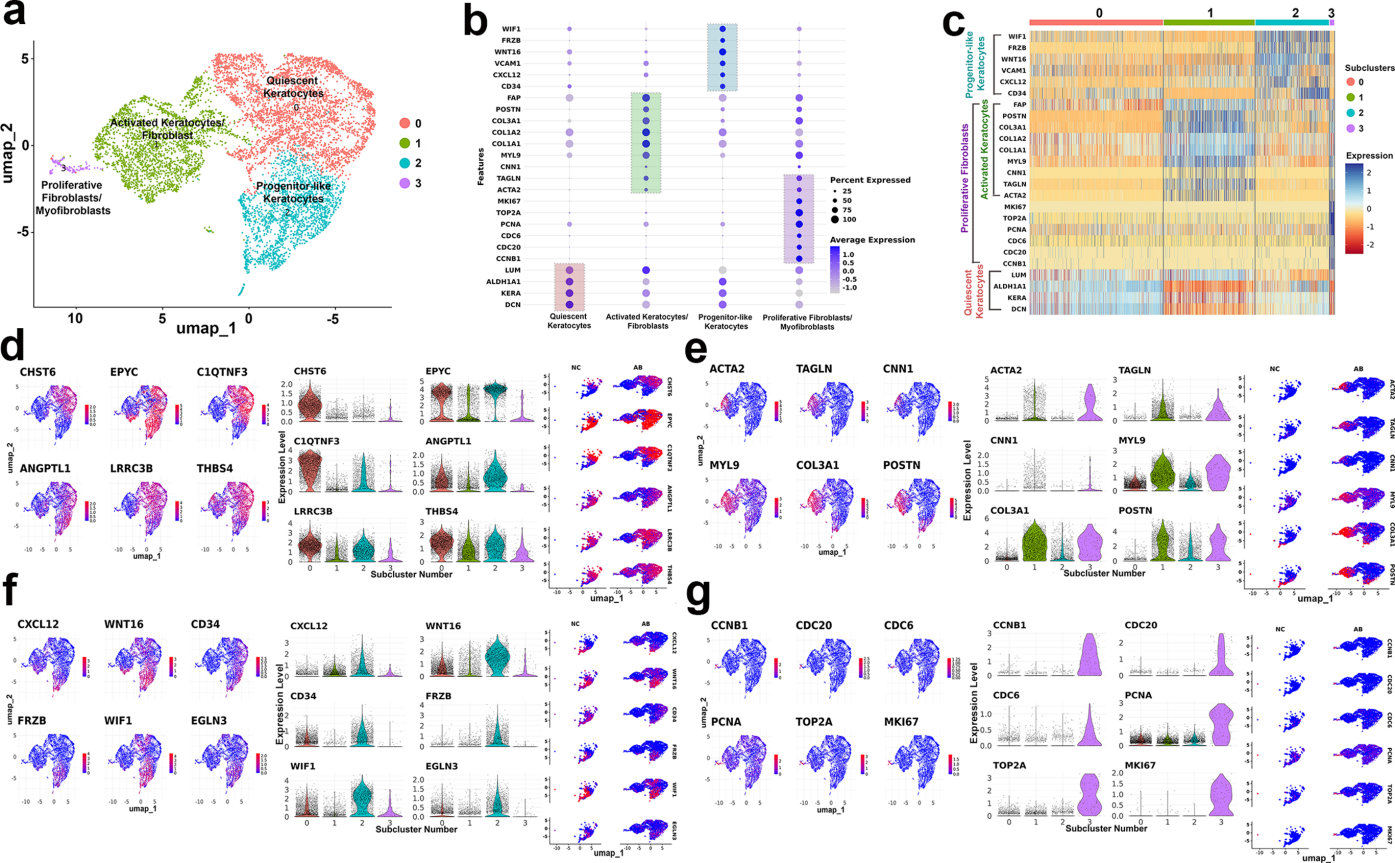
Subclustering of the ECM-remodeling cell cluster showing distinct cellular heterogenicity. **(a)** UMAP of ECM-remodeling cells showing four transcriptionally distinct subclusters (0–3) annotated as quiescent keratocytes, activated keratocytes/fibroblasts, progenitor-like keratocytes, and proliferative fibroblasts/myofibroblasts. **(b)** Dot plot of representative markers across subclusters; the dot size indicates the percentage of cells expressing the gene, and the color intensity indicates the average expression. **(c)** Heatmap of the top DEGs showing distinct differences among the four subclusters. **(d–g)** Feature maps, violin plots, and condition-split UMAPs (naïve/NC and fibrotic/AB at right) for canonical genes enriched in each state: **(d)** quiescent keratocytes (CHST6, EPYC, C1QTNF3, ANGPTL1, LRRC3B, and THBS4), **(e)** activated keratocytes/fibroblasts (ACTA2, TAGLN, CNN1, MYL9, COL3A1, and POSTN), **(f)** progenitor-like keratocytes (CXCL12, WNT16, CD34, FRZB, WIF1, and EGLN3), and **(g)** proliferative fibroblasts/myofibroblasts (CCNB1, CDC20, CDC6, PCNA, TOP2A, and MKI67). Color scales show log-normalized expression; violin plots depict gene expression distributions per subcluster.

The ECM remodeling (Cluster 2) is a central event in corneal fibrosis, subclustering analysis was performed to characterize transcriptional heterogeneity (Fig. 5a). This analysis identified four distinct stromal cell types, quiescent keratocytes (subcluster 0), activated keratocytes (subcluster 1), progenitor-like keratocytes (subcluster 2), and proliferative fibroblasts/myofibroblasts (subcluster 3) (Fig. 5a). Supplementary Table S4 shows the number of cells, whereas Supplementary Table S5 provides a list of differentially expressed genes within ECM-remodeling stromal cluster. The amount of change in unique genes across subclusters (Fig. 5b) and a heatmap of the top differentially expressed genes exhibiting distinct RNA profile in four cell subtypes (Fig. 5c) were determined. The distinct expression of marker genes across the subclusters using are shown using violon plots and separate UMAPs (Figs. 5d–g). A significant expression of key marker genes include CHST6, EPYC, C1QTNF3, ANGPTL1, LRRC3B, and THBS4 in quiescent keratocytes (Fig. 5d); ACTA2, TAGLN, CNN1, MYL9, COL3A1, and POSTN in activated keratocytes (Fig. 5e); CD34, WNT16, CXCL12, FRZB, WIF1, and EGLN3 in progenitor-like keratocytes (Fig. 5f); and CCNB1, CDC6, PCNA, MKI67, TOP2A, and CDC20 in proliferative fibroblasts/myofibroblast subcluster (Fig. 5g).

### Intercellular Communication Among ECM Remodeling Cells Has Demonstrated Signaling Diversity in Corneal Fibrosis

**Figure 6.**
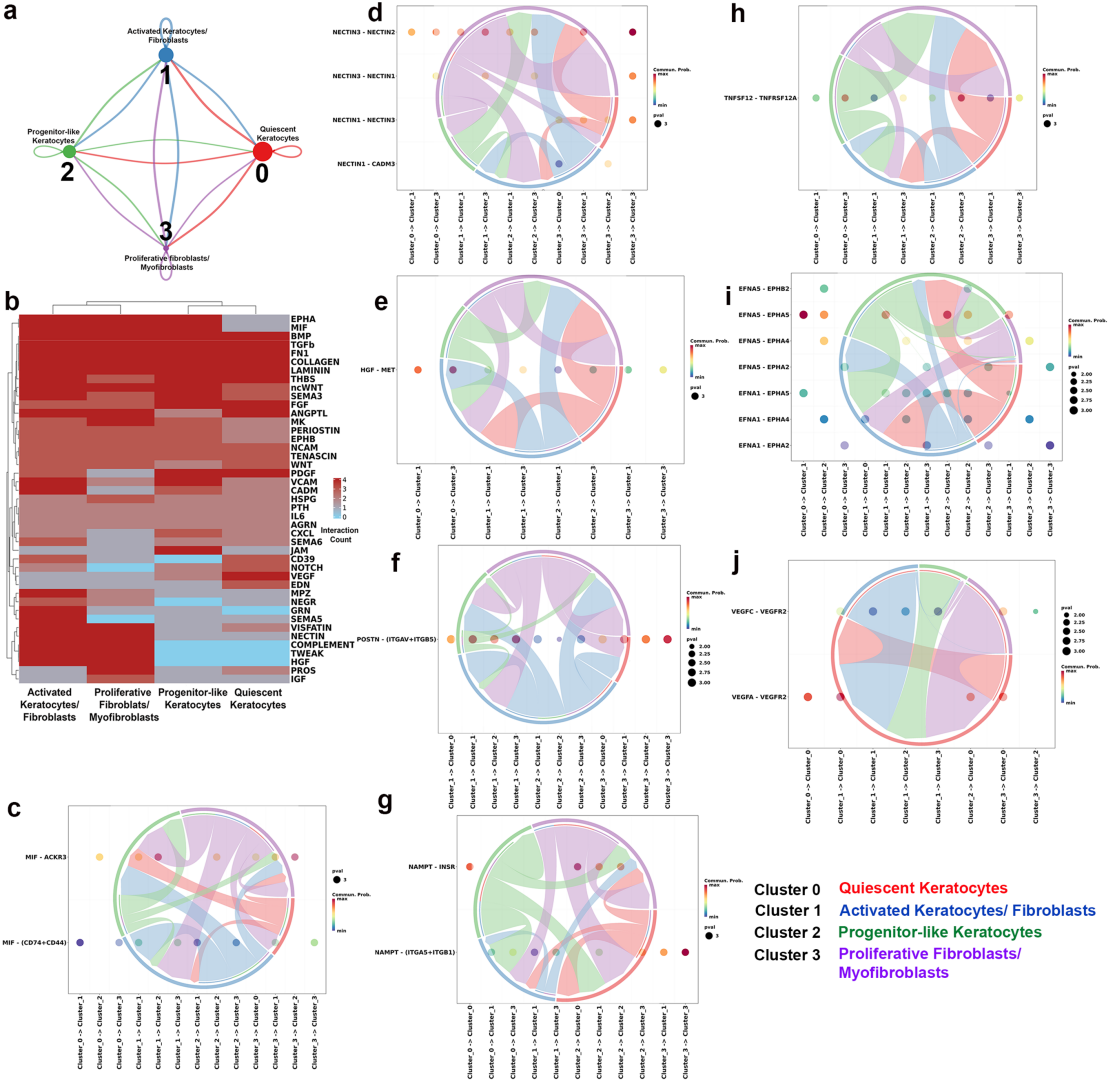
Intercellular communication within ECM-remodeling subclusters. **(a)** Network of inferred ligand–receptor exchanges among four stromal subclusters: 0-quiescent keratocytes, 1-activated keratocytes/fibroblasts, 2-progenitor-like keratocytes, and 3-proliferative fibroblasts/myofibroblasts; node size indicates the number of cells, and edge width denotes total signaling strength. **(b)** Heatmap of pathway-level enrichment across the four ECM-remodeling subclusters. **(c–j)** Chord diagrams with accompanying dot plots showing a representative subset of pathways; MIF **(c)**, NECTIN **(d)**, HGF **(e)**, POSTN **(f)**, NAMPT **(g)**, TWEAK/TNFSF12 **(h)**, EPHA **(i)**, and VEGF **(j)**, selected from the heatmap on the basis of differential expression and to cover distinct signaling families. The representative ligand–receptor pairs include MIF–CD74+CD44 or ACKR3, NECTIN3–NECTIN1/2, HGF–MET, POSTN–ITGAV+ITGB5, NAMPT–INSR or ITGA5+ITGB1, TNFSF12–TNFRSF12A, EFNA1/5–EPHA2/4/5, and VEGFA/VEGFC–VEGFR2/FLT4. The dot size reflects interaction strength, and the color intensity indicates communication probability.

Four transcriptionally distinct ECM remodeling cell subclusters demonstrating outbound signaling communications were identified (Fig. 6a). The subcluster-specific enrichment of key signaling pathways was uncovered by the intercellular ligand-receptor interactive expression heatmap (Fig. 6b). The differential gene expression and ligand-receptor connectivity among the four subclusters led to identification of eight novel pathways namely MIF, NECTIN, HGF, POSTN, NAMPT, TWEAK, EPH, and VEGFC. Pathway-resolved analysis further revealed directional and cell-specific signaling patterns via chord and dot plots (Figs. 6c–j). MIF signaling (Fig. 6c) demonstrated cellular communication among all subpopulations, mainly through MIF-ACKR3/CXCR7 and MIF-CD74+CD44 ligand-receptor interactions. NECTIN signaling (Fig. 6d) was driven largely via NECTIN3-NECTIN1/2 ligand-receptor interactions. HGF signaling was more prominent in activated keratocyte/fibroblast and proliferative fibroblast/myofibroblast subclusters via HGF-MET signaling (Fig. 6e). POSTN signaling (Fig. 6f) was broadly distributed across all stromal cell subtypes via POSTN-(ITGAV+ITGB5) signaling except the quiescent keratocytes. NAMPT (also known as VISFATIN) signaling (Fig. 6g) was distributed predominantly in the activated keratocyte/fibroblast and proliferative fibroblast/myofibroblast subclusters, mostly via the NAMPT-INSR and NAMPT-(ITGA5+ITGB1) ligand-receptor pairs TWEAK (also known as TNFSF12) signaling (Fig. 6h) was also largely present in the activated keratocyte/ fibroblast and proliferative fibroblast/myofibroblast subclusters driven by the TNFSF12-TNFRSF12A ligand-receptor interaction. EPHA signaling (Fig. 6i) appeared to be distributed across all stromal subtypes, involving EFNA1/5 with EPHA2/4/5 receptors. VEGF signaling (Fig. 6j) appeared to be disseminated via VEGFA-VEGFR2 and VEGFC-VEGFR2 interactions.

### Verification of ECM Remodeling Signatures and Cellular Signaling

**Figure 7.**
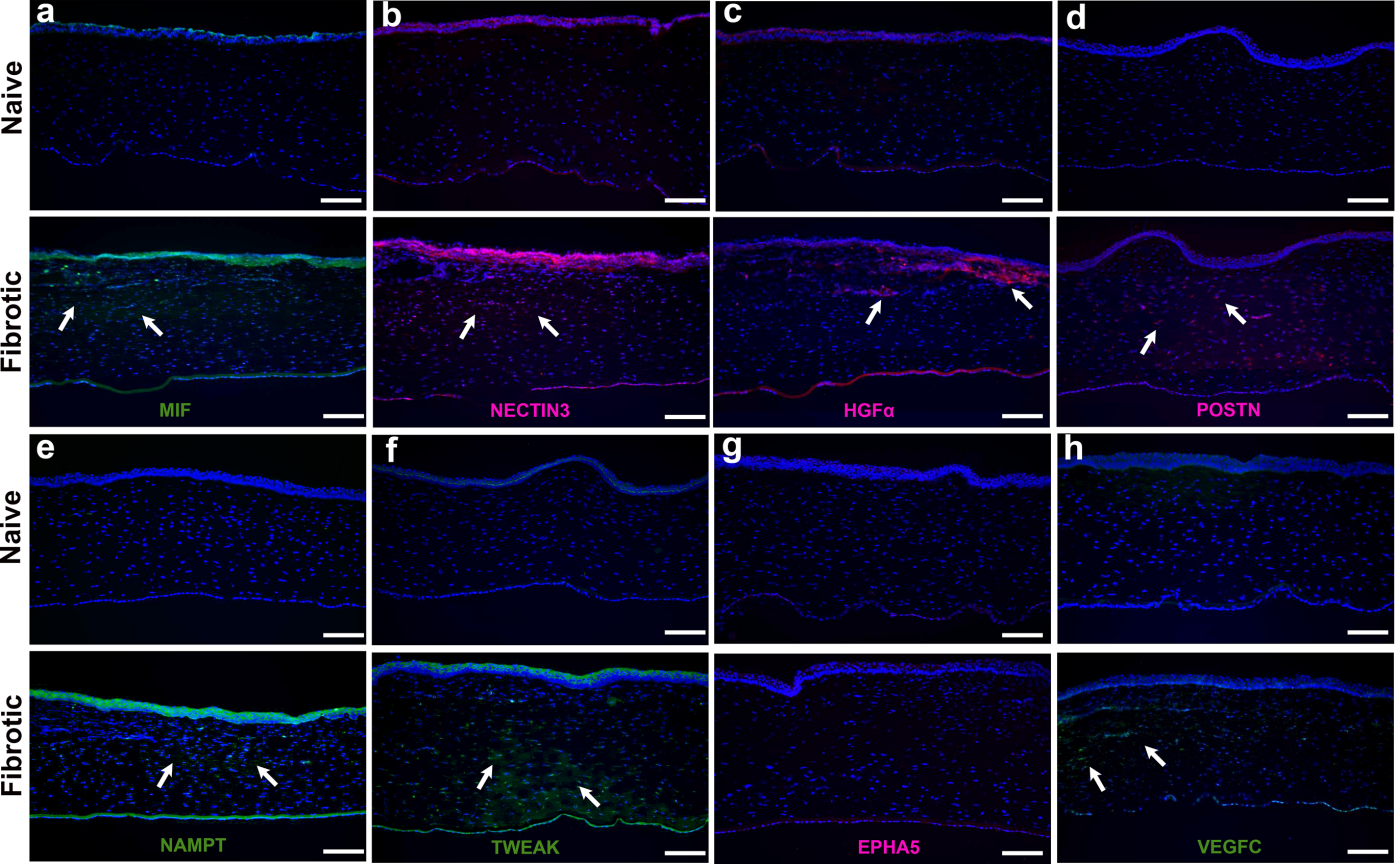
Protein-level assessment of selected mediators showing spatial upregulation in fibrotic tissue. **(a–d)** MIF (*green*), NECTIN3 (*red*), HGFα (*red*), and POSTN (*red*) are shown for naïve tissue (*top*) and fibrotic tissue (*bottom*). Fibrotic corneas show increased protein expression in the epithelium and anterior stroma, with POSTN being expressed mainly in the stroma (*arrows*). **(e–h)** NAMPT (*green*), TWEAK (*green*), EPHA5 (*red*), and VEGF-C (*green*) are shown for naïve tissue (*top*) and fibrotic tissue (*bottom*). Increased protein expression of NAMPT and TWEAK in the epithelial and stromal layers, VEGFC showing focal stromal expression (*arrows*), and EPHA5 remains undetectable. Nuclei were counterstained with DAPI; *n* = 3 biological replicates per group. *Scale* *bar*: 100 µm.

Whether transcriptomic predictions from intercellular communication in ECM-remodeling cell subclusters (Fig. 6) apply at protein levels, the expression of MIF, NECTIN, HGF, POSTN, NAMPT, TWEAK, EPH, and VEGFC genes in naïve and fibrotic rabbit corneas was analyzed by immunofluorescence (Fig. 7). MIF (Fig. 7a) and NECTIN3 (Fig. 7b) expression was significantly higher in epithelium and anterior stroma of fibrotic cornea than the naïve. Compared the naïve cornea, HGFαwas prominently localized to the basal epithelium and anterior stroma of the fibrotic cornea (Fig. 7c). POSTN expression was widespread in stroma of the fibrotic cornea while it was absent in naïve cornea (Fig. 7d). NAMPT (Fig. 7e) and TWEAK (Fig. 7f) expression were markedly upregulated in epithelial and stromal layers of the fibrotic cornea but was absent in the naïve cornea. Interestingly, EPHA5 protein expression was not detected in naïve and fibrotic corneas (Fig. 7g) despite its predicted involvement in intersubcluster signaling. VEGFC (Fig. 7h) was mildly expressed in the stroma of fibrotic corneas only and not in the naïve cornea.

### Pseudotime-Guided Mapping of ECM-Remodeling Cellular State Transitions and Stage-Specific Pathway Enrichment

**Figure 8.**
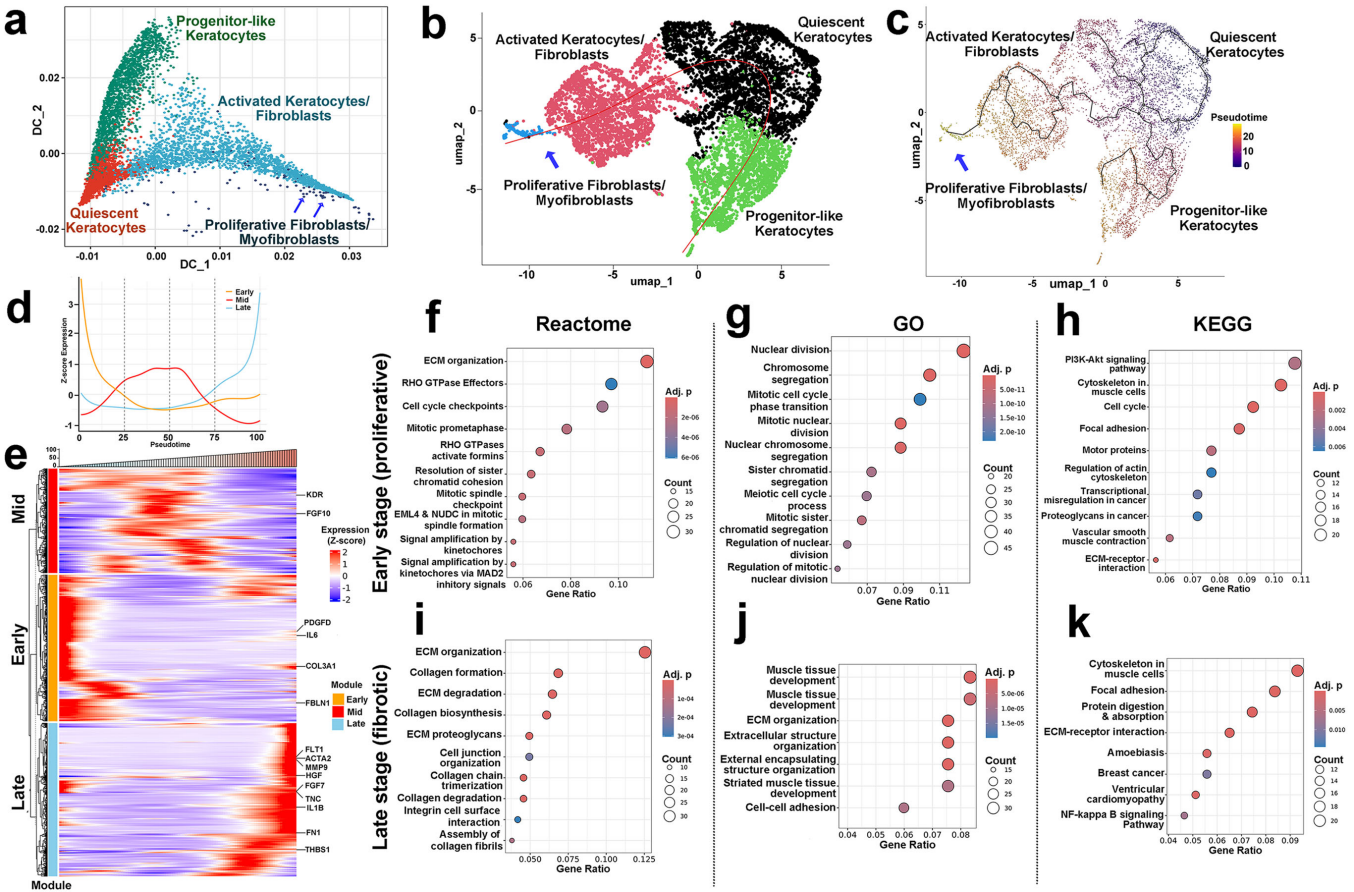
Trajectory inference and temporal pathway enrichment within the ECM-remodeling niche. **(a)** Diffusion map positioning four stromal states along a bifurcating continuum that begins with quiescent keratocytes and diverges toward progenitor-like keratocytes and proliferative fibroblasts/myofibroblasts; activated keratocytes/fibroblasts occupy intermediate positions. **(b)** UMAP of the same cells colored by subcluster identity; *red curve* indicates the bifurcating trajectory from keratocytes (root). **(c)** Slingshot trajectories and pseudotime overlaid on UMAP. **(d)** Smoothed mean expression profiles for early, middle, and late gene modules plotted across pseudotime. **(e)** Heatmap of Z score–scaled genes ordered by pseudotime and grouped by the temporal module; selected genes are annotated. **(f–h)** Enrichment of early module genes in the reactome **(f)**, GO biological process **(g)**, and KEGG **(h)**, depicting the cell cycle, mitotic, and PI3K–AKT pathways. **(i–k)** Enrichment of late module genes in the reactome **(i)**, GO biological process **(j)**, and KEGG **(k)**, showing ECM organization along with collagen formation, focal adhesion, and ECM–receptor interaction. The point size denotes the number of genes contributing to each term; the color represents the adjusted *P* value; and the x-axis represents the gene ratio.

Pseudotime trajectory inference via the Slingshot algorithm was performed to resolve the temporal dynamics within ECM remodeling cells. The diffusion map (Fig. 8a) and UMAP embedding (Figs. 8b, 8c) identified a continuous landscape of stromal cell subpopulations progressing from quiescent keratocytes towards proliferative fibroblasts/ myofibroblasts and progenitor-like cellular states. The inferred trajectory indicated bidirectional progression along pseudotime. The gene expression profiles were smoothed to stratify the genes into early, intermediate, and late transcriptional modules (Fig. 8d), with distinct Z score patterns visualized in a module-specific heatmap (Fig. 8e). Pathway enrichment via Reactome, GO, and KEGG revealed significant enrichment of early-stage pathways such as ECM organization, nuclear division, mitotic progression, cell cycle regulation, and PI3K‒Akt signaling (Figs. 8f–h), which was consistent with a proliferative phenotype. ECM‐related pathways displayed modest early activation and a dominant late surge characterized by ECM organization, collagen formation, focal adhesion, and ECM-receptor interaction (Figs. 8i–k). Supplementary Table S6 presents enrichment analyses for early- and late-stage pseudotime in ECM-remodeling.

## Discussion

Single-cell RNA-seq allows precise information of cellular heterogeneity, cell-to-cell variations, intercellular dynamics, and measurement of transcript expression of cells within a specific microenvironment. After insult, cornea exhibits hallmark features of fibrosis characterized by opacity, architectural disarray, alterations in cellular make-up including myofibroblast formation, and aberrant ECM remodeling (Fig. 1). This study presents a comprehensive single-cell atlas demonstrating cellular heterogeneity, ligand-receptor connectivity, transcriptional trajectories, intercellular communication, distinct landscape of cell populations, temporal dynamics in ECM remodeling, and interactive framework of genes in fibrotic and naïve corneas in vivo*.*

Transcriptionally distinct 14 cell populations encompassing all three layers of the cornea, epithelium, stroma, and endothelium, along with neovascular, neural, and immune compartments were recognized (Fig. 2). In the epithelium, the basal layer is expanded with the expression of markers such as CDH3, SOX9, KRT15, and LAMB3, a pattern compatible with an activated, repair-oriented state. Compared with the naïve cornea, the fibrotic stromal compartment showed contraction of quiescent keratocytes alongside expansion of ECM remodeling, activated fibroblasts, and persistent myofibroblasts suggesting a shift from ECM maintenance to ECM production and contractility. Together, the epithelial and stromal changes indicated that corneal fibrosis involves a dynamic crosstalk with epithelial cell and stromal matrix responses in parallel and potentially influencing each other through intercellular signaling. Additionally, the vascular and immune compartments, which align with the inflammatory and neovascular milieu observed in the fibrotic corneal tissue, were more prominent. These results provide a cellular roadmap of corneal remodeling at single-cell resolution and establish a framework for investigating injury-induced lineage cellular transitions.

Inter-cluster communication mapping across the fourteen cell types resulted in a dense but uneven network. The intercellular communication centered on EMT-like cells, ECM-remodeling cells, keratocytes, and basal corneal epithelium indicating the role and coordination of factors across epithelial and stromal niches (Figs. 3a–c). The detection of broadly distributed collagen signaling was consistent with aberrant ECM remodeling and communication as a common substrate during fibrosis, whereas TGFβ, JAMA, and LAMININ signals are more selective, especially with differentiated epithelium (cluster 6), which exhibits limited signaling through these three pathways and remains relatively communicative‒quiescent during the observed phase of fibrosis and wound healing (Figs. 3d–g).

The protein expression of COL1A1, TGFβ1, JAMA, and LAMININ5 also aligned with the fibrotic network structure. Higher COL1A1 expression suggesting increased collagen deposition due to irregular ECM remodeling, while high expression of TGFβ1 is consistent with its central role in activating quiescent keratocyte and activated fibroblast, ECM synthesis, and myofibroblast production during fibrosis (Figs. 3h, 3i).^26^ JAMA and LAMININ5 extended along epithelial-stromal junctions in the fibrotic cornea (Figs. 3j, 3k) highlighted the emergence of matrix-integrin and adhesion-based signaling loops during aberrant wound healing in fibrotic condition and led to a notion that signaling is focused in an area where basal epithelial and stromal populations interact.

Furthermore, targeted analysis within the stromal cellular microenvironment revealed the activation of canonical fibrotic signaling pathways, including the TGFβ, PDGF, and VEGF pathways (Fig. 4). This aligned well with earlier studies from our and other corneal researchers.^27^^–^^31^ In vivo scRNA-seq showing TGFβ signaling targeted a broad spectrum of recipient clusters, including vascular and activated keratocytes (Fig. 4b). This observation was corroborated by the protein expression of TGFβ1 (Fig. 3i) and TGFβ2 (Fig. 4h) via the TGFBR1/2 and ACVR1 receptors in the sender and receiver populations (Fig. 4e) highlighted the spatial relevance to fibrotic remodeling. Similarly, PDGF signaling emerged as a prominent pathway, with PDGFA/B/C/D–PDGFRA/B interactions mediating crosstalk between persistent myofibroblasts and ECM-remodeling clusters (Figs. 4c, 4f). In parallel, the VEGF pathway, particularly the VEGFA–VEGFR1/2 pair, was enriched in interactions primarily involving vascular cell clusters (Figs. 4d, 4g), and this finding was supported by VEGFA protein expression in the anterior stroma of fibrotic corneas (Fig. 4j). Tissue staining was concordant with this structure, with increased TGFβ1/2 (Figs. 3i, 4h), PDGFA (Fig. 4i), and VEGFA signals (Fig. 4j) in the injury-adjacent stroma was in accordance with growth factor-driven activation, proliferation, ECM deposition, and vascular remodeling in corneal wound healing.^32^^–^^35^

Outside the stroma, communication hubs appear in the basal and EMT-like epithelium, with activity in the FGF, WNT, TGFβ, and NOTCH pathways that is consistent with barrier repair (Supplementary Fig. S1a). As stated earlier, the differentiated epithelial layer, by contrast, showed limited participation in these pathways, which reaffirms that dynamic exchange was concentrated at the basal epithelial-anterior stromal interface. Interactions between nerve or Schwann cells and immune populations further suggest a neuroimmune contribution that may fine-tune the wound milieu (Supplementary Fig. S1b).

Subclustering of ECM-remodeling cells (cluster 2) resolved four discrete cellular states (i.e., quiescent keratocytes, activated keratocytes/fibroblasts, progenitor-like keratocytes and proliferative fibroblasts/myofibroblasts) (Figs. 5a–c). Each state displayed a unique transcriptional fingerprint. Quiescent keratocytes maintained canonical corneal matrix genes, whereas activated fibroblasts upregulated contractile and fibrotic signatures. Another subcluster expressed classic progenitor-like cell markers, and the proliferative fibroblast/myofibroblast cluster exhibited brisk cell cycle activity, including the expression of mitotic checkpoint genes (Figs. 5d–g). This hierarchical stratification highlights the dynamic state transitions, phenotypic plasticity, and reparative potential within the ECM-remodeling cell niche during corneal fibrosis. A quantitative comparison revealed that fibrosis dramatically expanded the proliferative, activated fibroblast or myofibroblast pools while leaving the progenitor-like cluster almost numerically intact (Supplementary Table S4). This data prompted us to predict that ECM remodeling is fueled by two converging processes, cell cycle–driven amplification of fibroblasts and myofibroblast maturation, both of which are possibly fed by a retained progenitor-like niche or cell types. Therapeutically, this hierarchy argues for dual-phase intervention, which involves targeting the proliferative surge followed by inhibition to prevent contractile maturation while sparing the progenitor pool needed for long-term regeneration. Nevertheless, this evolving concept needs additional experimental evidence.

Further investigation of intercellular signaling among the four ECM-remodeling cell subclusters showed distinct transcriptional divergence (Fig. 6). On the basis of the differentially expressed pathways (Fig. 6b), chord plot analysis and ligand‒receptor interactions (6c-j), eight key mediators (ligands), namely, MIF, NECTIN3, HGFα, POSTN, NAMPT, TWEAK, EPHA5, and VEGFC, were identified. The predicted mediators were evaluated at the protein level via immunofluorescence staining across naïve and fibrotic corneal tissues (Fig. 7). This evaluation suggests that transcriptional predictions from the ECM-remodeling subclusters converge at the tissue level on a networked remodeling program rather than a single driver. The protein expression of MIF (inflammatory cue),^26^ NAMPT (metabolic/cytokine-like signal), and TWEAK (TNF-superfamily ligand), although acting differently, converge on profibrotic signaling associated with fibroblast activation and ECM turnover. NECTIN3 (cell-cell junctions)^36^^,^^37^ and POSTN (matricellular, integrin-potentiating adhesion)^38^^,^^39^ map to complementary adhesion modules and seem consistent with scar consolidation. The increased expression of HGFα in fibrotic corneas supports epithelial-stromal communication, whereas EPHA5 was not detected in fibrotic tissue.^40^ Thus EPH family guidance signaling remains transcriptionally predicted rather than protein confirmed in corneal tissue. The presence of VEGFC signals along with VEGFA (Fig. 4j) suggests vascular growth signaling which occurs during fibrosis.^41^^,^^42^ Because immunostaining was performed on whole corneal sections, these findings provide spatial corroboration of pathway engagement rather than assignment to specific ECM subclusters or cell-cell pairs. Even so, the pattern supports network-level targeting. This data encouraged us to postulate that prioritizing targeting of inflammatory/metabolic mediators during cellular transition, adhesion, and ECM remodeling phases in an injured cornea may avoid over-attribution to any single ligand-receptor pathway. Our future studies will investigate this concept.

Trajectory reconstruction placed the four ECM remodeling subclusters along a diffusion axis that initiates with quiescent keratocytes, progresses through a population of activated keratocytes/fibroblasts, and bifurcates toward two terminal states: a cluster of progenitor-like keratocytes and a population of proliferative fibroblasts/myofibroblasts (Figs. 8a, 8b). This transcriptional trajectory reflects a canonical stromal activation and remodeling process in which resting keratocytes first undergo phenotypic activation and then diverge either toward regenerative progenitor-like cells or become fibrotic cells with contractile phenotype. Pseudotime analysis (Fig. 8c) via the Slingshot algorithm supported our hypothesis, revealing a temporal gradient beginning with keratocytes and increasing toward both bifurcating endpoints. However, trajectory directions reflect transcriptional ordering at a single time point rather than proven fate transitions; multi-timepoint sampling will refine lineage directionality. Nevertheless, these findings suggest that, by day 14 after injury, the corneal stroma remains actively engaged in various wound healing cellular transformation programs distributed along various fibrotic and regenerative trajectories.

The enrichment patterns indicate a two-phase remodeling model. Early modules are dominated by proliferative/priming pathways, including cell cycle/mitotic and PI3K–AKT signaling, which are consistent with the stromal expansion that precedes lineage bifurcation (Figs. 8f–h). ECM and adhesion pathways seem biphasic, showing a modest early rise but late dominance, aligning with a shift from provisional turnover to matrix consolidation (Figs. 8i–k). This observed bifurcation suggests that proliferative pressure facilitates cellular state transitions, whereas adhesion and matrix pathways stabilize the fibrotic endpoint, although a progenitor-like cell remains transcriptionally distinct and potentially regenerative. It is worth noting that these inferences are correlative because pseudotime arranges cells into a likely progression using one-time-point data. The lack of multiple time points and targeted perturbation experiments limits definitive inferences about when these changes occur.

Overall, this study provides a high-resolution single-cell atlas of fibrotic corneas that outlines the cellular composition, framework, and signaling features of stromal remodeling in vivo along with epithelial changes and ECM remodeling in stromal cell populations (quiescent keratocyte, activated keratocyte, progenitor-like keratocyte and proliferative-fibroblast/myofibroblast). Multiple ligand-receptor mediators of stromal communication were identified at transcriptional level, whereas the top eight were studied at the protein level in corneal tissue, which indicated coordinated inflammatory, adhesion, and trophic signaling during matrix remodeling. Transcriptional profiles resolved a bifurcating stromal continuum: one branch aligned with fibroblast activation/myofibroblast features whereas the other retained quiescent or progenitor-like keratocyte characteristics. Additionally, this study affords an integrative framework of intercellular communication, trajectory inference, and tissue-level protein expression mediating corneal fibrosis and lays the foundation for the development of mechanism-based anti-fibrotic strategies.

## Supplementary Material

Supplement 1

Supplement 2

Supplement 3

Supplement 4

Supplement 5

Supplement 6

Supplement 7
