# Supplementary material for: Single-Cell Transcriptomics Shows Cellular Heterogeneity, Intercellular Communication, and Extracellular Matrix Remodeling in Corneal Fibrosis In Vivo
Source: Invest Ophthalmol Vis Sci. 2025 Oct 28;66(13):48. doi: 10.1167/iovs.66.13.48 (PMC12577772; doi:10.1167/iovs.66.13.48)
Supplement: Supplement 1 [file iovs-66-13-48_s001.docx]

**
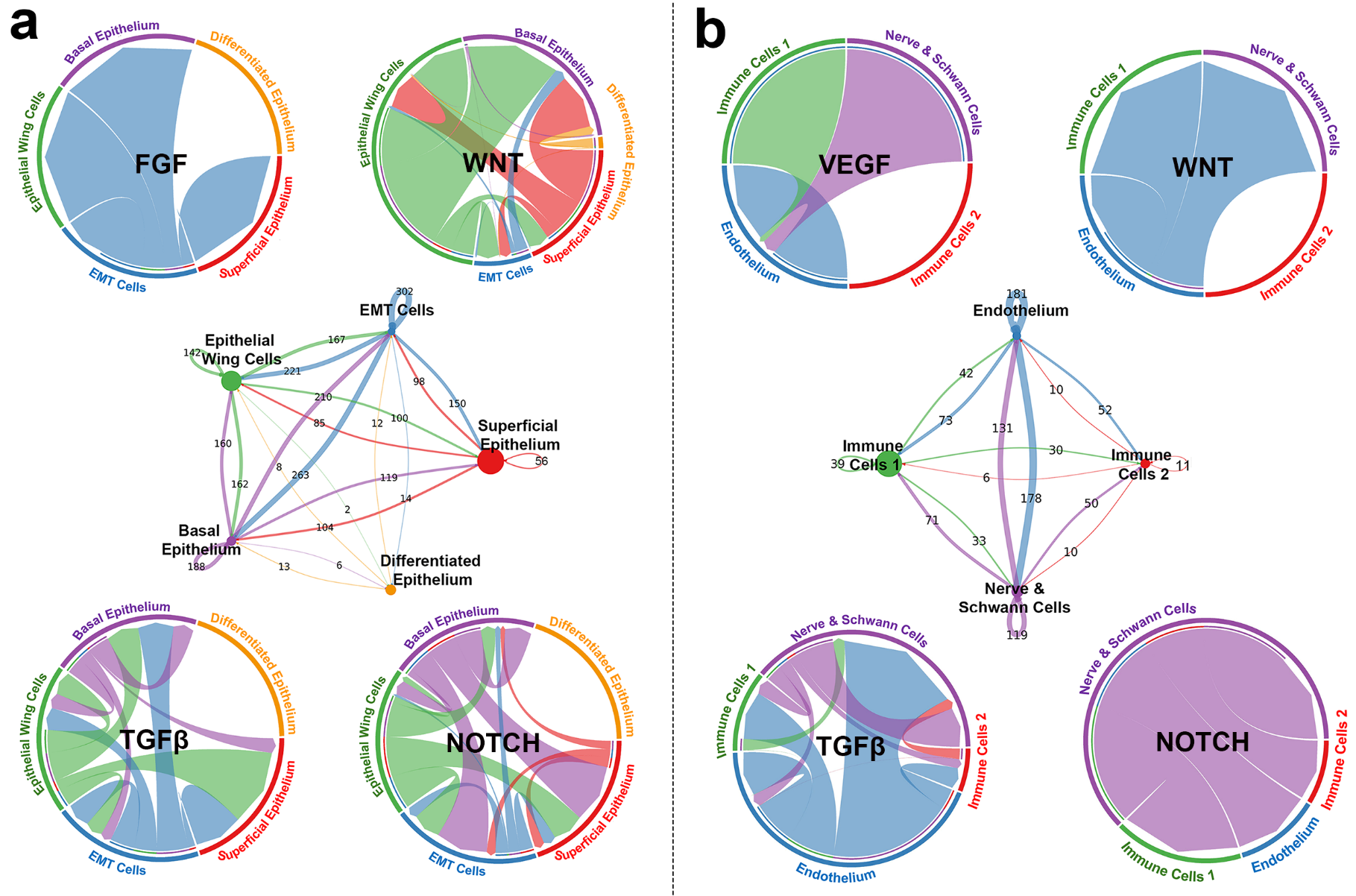
**

**FIGURE S1. Intercellular communication across nonstromal corneal layers.** (a) Communication network and chord diagrams depicting inferred intercellular signaling among five corneal epithelial subtypes: EMT cells, epithelial wing cells, basal epithelium, superficial epithelium, and differentiated epithelium. CellChat analysis highlights key signaling pathways, including TGFβ, NOTCH, WNT, and FGF, revealing dominant communication routes, primarily from EMT cells to other epithelial populations. (b) Corresponding analysis of endothelial, nerve/Schwann, and immune cell subsets (immune 1 and immune 2) revealed dense intercellular signaling via VEGF, WNT, TGFβ, and NOTCH pathways, with endothelial cells and Schwann cells serving as central hubs. Edge thickness in network plots corresponds to the number of inferred interactions; chord diagrams illustrate the directionality and strength of individual signaling pathways.

**Table S1.** The cell barcode annotation table lists the corresponding sample origin and transcriptionally defined cluster assignment for each cell.

**Table S2**. Predicted corneal cell types with considered marker genes and supporting references used for cluster annotation.

**Table S3.** Cluster-specific DEGs identified from single-cell transcriptomes of rabbit corneas. The data included log2-fold changes, adjusted p values, and cluster-level gene enrichment metrics for the annotated cell types.

**Table S4.** Number of cells from each sample assigned to stromal subclusters following reclustering.

**Table S5.** Differentially expressed genes for each subcluster identified within the ECM-remodeling stromal cluster.

**Table S6.** Reactome, GO, and KEGG pathway enrichment analyses for early-stage and late-stage pseudotime gene modules identified in ECM-remodeling cells. Enrichment was performed separately for each temporal module to reveal distinct biological programs associated with proliferation (early) and fibrosis (late).
